# Characterization of a Novel ArsR-Like Regulator Encoded by Rv2034 in *Mycobacterium tuberculosis*

**DOI:** 10.1371/journal.pone.0036255

**Published:** 2012-04-27

**Authors:** Chun-hui Gao, Min Yang, Zheng-Guo He

**Affiliations:** National Key Laboratory of Agricultural Microbiology, Center for Proteomics Research, College of Life Science and Technology, Huazhong Agricultural University, Wuhan, China; University of Padova, Medical School, Italy

## Abstract

The genome of *Mycobacterium tuberculosis*, the causative agent of tuberculosis, encodes a large number of putative transcriptional regulators. However, the identity and target genes of only a few of them have been clearly identified to date. In a recent study, the ArsR family regulator Rv2034 was characterized as a novel positive regulator of *phoP*. In the current study, we characterized the auto-repressive capabilities of Rv2034 and identified several residues in the protein critical for its DNA binding activities. We also provide evidence that Rv2034 forms dimers *in vitro*. Furthermore, by using DNaseI footprinting assays, a palindromic sequence was identified as its binding site. Notably, we found that the *dosR* promoter region contains the binding motif for Rv2034, and that Rv2034 positively regulates the expression of the *dosR* gene. The potential roles of Rv2034 in the regulation of lipid metabolism and hypoxic adaptation are discussed.

## Introduction

*Mycobacterium tuberculosis*, the causative agent of tuberculosis, is a leading cause of death worldwide [1]. One-third of the global population is latently infected with this pathogen and millions die each year due to active tuberculosis [2]. The success of *M. tuberculosis* bacilli is linked to its ability to adapt to hypoxia and persist within humans for long periods without causing any overt disease symptoms [3]–[6]. Uncovering the mechanisms that regulate its adaptability and persistence is therefore of great clinical and public health significance.

Multiple studies *in vitro* and *in vivo* have indicated that DosR is the key regulator that mediates hypoxic responses in *M. tuberculosis* [3], [6], [7]. However, a recent study claimed that only the initial hypoxic response genes are regulated by dosR [8] while the regulatory mechanism of enduring hypoxic response genes remains unclear.

The ArsR family regulator is widely expressed in many bacterial or archaeal species and is highly abundant in some of them [9]. For example, *Corynebacterium gutamicum* and *Streptomyces coelicolor* have 12 and 23 copies of ArsR homology, respectively [9]. *M. tuberculosis* has 12 ArsR homologies [10]. Generally, the ArsR transcriptional regulators in many bacterial species or archeae act as metal sensors that repress gene expression during peacetime, and release from the promoters to de-repress gene expression when metal ions become abundant [11]–[15]. However, some of the ArsR-type regulators have also been found to be involved in bacterial pathogenesis. In *Vibrio cholerae*, the regulator HlyU regulates expression of the virulence determinant HlyA [16]. In *Vibrio vulnificus*, the HlyU protein upregulates the expression of the essential virulence factor *rtxA1* [17], [18]. Besides, the SloR protein from *Streptococcus mutans* [19] and the PagR protein from *Bacillus anthracis* [20] have both been shown to be essential for bacterial pathogenesis. These findings suggest that ArsR regulators may also act as regulators of bacterial pathogenesis.

To our knowledge, four of the twelve ArsR family regulators in *M. tuberculosis* have been characterized as metal sensors: KmtR (encoded by Rv0827c) and NmtR (encode by Rv3744) sense nickel-cobalt [21], [22], CmtR (encoded by Rv1994c) senses cadmium and lead [12], [23], and SmtB (encoded by Rv2358) senses zinc [21], [24]. Among them, CmtR and SmtB are capable of self-regulation [12], [23], [24]. In our previous study, we characterized the ArsR family regulator Rv2034 as a novel positive regulator of *phoP* and *groEL2* [10]. However, the potential of Rv2034 for self-regulation was unclear.

In the present study, we have characterized the ability of Rv2034 for auto-regulation in *M. tuberculosis*. The binding site of Rv2034 to its promoter DNA was mapped out and some residues critical in Rv2034 for DNA binding activity were also identified. Furthermore, a novel gene target of Rv2034, the *dosR* gene, was identified. Based on our analysis of its target genes, we conclude that Rv2034 is a transcriptional regulator involved in the regulation of lipid metabolism and hypoxic response in *M. tuberculosis*.

## Results

### Rv2034 specifically binds to its own promoter DNA sequence

**Figure 1:**
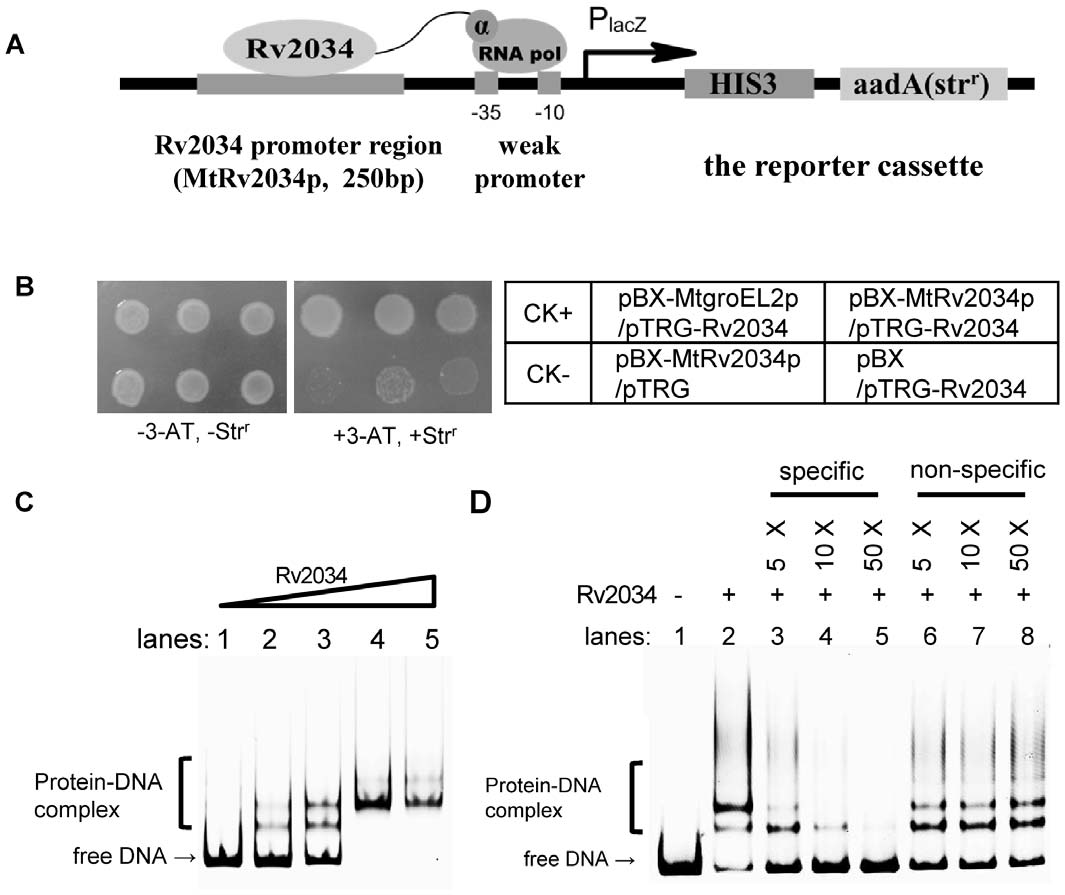
Self-regulation of Rv2034. (**A**) The putative promoter region of Rv2034 was cloned upstream of the *HIS3-aadA* reporter genes in the bacterial one-hybrid reporter vector pBXcmT, as described in the Materials and Methods section. (**B**) Bacterial one-hybrid assays for the interaction between Rv2034 and its promoter DNA. Each unit in the right panel represents the corresponding co-transformant in the plates. Co-transformants containing pBX-MthspXp and pTRG-Rv3133c were used as positive controls (CK+), while co-transformants containing pBX-MthspXp and pTRG-Rv3133cΔC were used as negative controls (CK−). Left panel: bacterial one-hybrid plates. Right panel: an outline of the plates in the left panel. Each unit represents the corresponding co-transformant in the plates. (**C**) EMSA assays for the binding of Rv2034 to the Rv2034p promoter DNA. The promoter DNA was derived from the region upstream of the Rv2034 coding region and included the first three nucleotides of the Rv2034 coding sequence. The DNA substrate was labeled by fluorescein isothiocyanate (FITC) and co-incubated with various amounts of the his-tagged Rv2034 protein (0, 0.1, 0.2, 0.4, and 0.8 µM). (**D**) The competition assays. Unlabeled cold Rv2034p DNA substrates or non-specific DNA substrates (the coding sequence of green fluorescent protein) were tested for their ability to compete with the labeled Rv2034p promoter DNA. Only the specific Rv2034p, and not the non-specific DNA, could competitively inhibit the binding of Rv2034 to the labeled Rv2034p promoter DNA substrate.

We used a bacterial one-hybrid system [25], which detects protein-DNA interactions based on transcriptional activation of the reporter genes *HIS3* and *aadA*, to detect interaction of Rv2034 with its promoter. The coding sequence of Rv2034 was cloned into the pTRG vector. The putative promoter region of Rv2034 was cloned upstream of *HIS3-aadA* reporter genes of the bacterial one-hybrid reporter vector pBXcmT [25] as shown in Figure 1A. Growth of co-transformants containing the pTRG and the pBXcmT vectors were monitored in plates with (+3-AT, +Str^r^) or without (−3-AT, −Str^r^) the screening media (see Materials and Methods section). If the regulator (in the pTRG vector) is able to activate the promoter (in the pBXcmT vector), the reporter genes should be induced and the transformed cells therefore grow well in the selective plate. The co-transformants containing pBX-Mt2034p/pTRG-Rv2034 grew very well in the screening medium, as shown in Figure 1B. The positive control composed of co-transformants with pBX-Mt2031p/pTRG-Rv3133c (CK+) and co-transformants with pBX-MtgroEL2p/pTRG-Rv2034, which has been characterized in a previous work [10], also grew well in the screening medium (Figure 1B). By contrast, no growth was observed for the negative control composed of co-transformants with pBX-Mt2031p/pTRG-Rv3133cΔC (CK−). Besides, only slight growth was observed for their self-activated controls (Figure 1B).

To further confirm the interaction between Rv2034 and its promoter, an EMSA assay was performed. The promoter region of Rv2034 consists of ∼250 nucleotides situated upstream of its start codon. As shown in Figure 1C, when 3 nM of the Rv2034 promoter DNA substrates were co-incubated with increasing amounts of Rv2034 (0, 0.1, 0.2, 0.4, 0.8 µM), clear shifts in the bands were clearly observed (Figure 1C, lanes 2–5). A competition assay was performed subsequently. Unlabeled cold Rv2034 promoter DNA or unspecific GFP coding DNA were tested for their ability to compete with the labeled Rv2034 promoter DNA (the GFP coding DNA was amplified using pFPV1 as template [26], see Table S3 for more information). As shown in Figure 1D, cold Rv2034 promoter DNA, but not GFP coding DNA, could competitively inhibit the binding of Rv2034 to the labeled Rv2034 promoter DNA substrate. These results indicate that Rv2034 can specifically bind its own putative promoter region.

### Rv2034 auto-represses its expression

**Figure 2:**
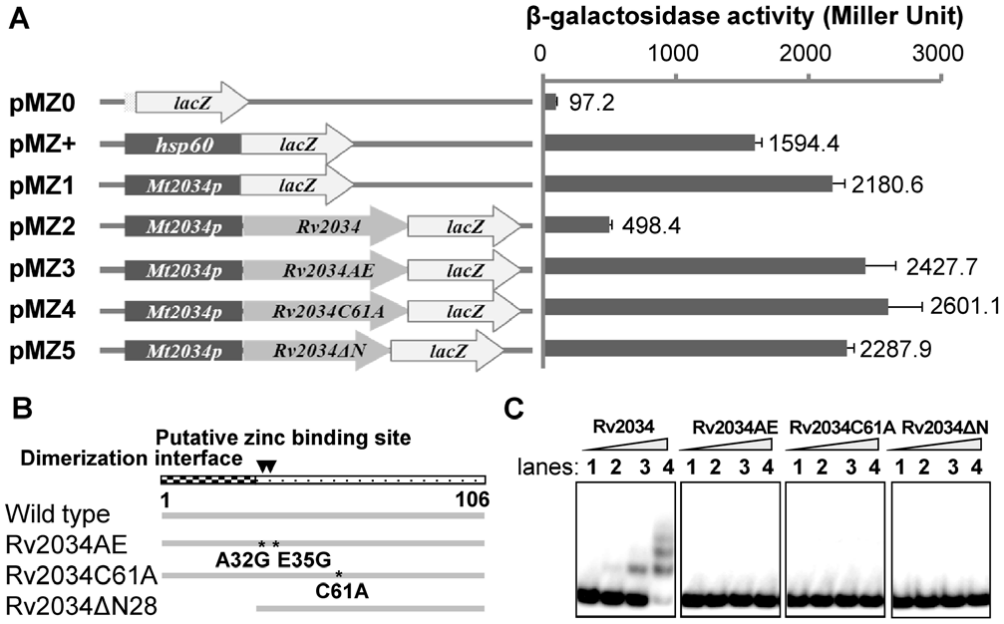
DNA-binding activities of Rv2034 wild type and mutant proteins. (**A**) The effects of Rv2034 and its mutant variants on gene expression were measured by β-galactosidase activity assays. The names of *lacZ*-fused plasmids are listed in the left panel, and the elements differing among the plasmids are shown in the middle panel. β-galactosidase activities are presented as Miller units in the right panel. The values reported are the averages of three independent experiments. Error bars represent standard deviations. (**B**) Diagram illustrating Rv2034 variants tested in our assays. The dimerization interface and putative zinc binding sites are illustrated. The Rv2034AE (A32G, E35G) and Rv2034C61A variants were produced by site-directed mutagenesis as described in Materials and Methods. The Rv2034ΔN28 variant was produced by deleting the first 28 amino acid residues from the N-terminal of Rv2034. (**C**) EMSA assays for the binding of Rv2034 variants to the Rv2034p promoter DNA. The mutant variants of Rv2034 were purified as described in Materials and Methods and co-incubated with the DNA substrate labeled with [γ- ^32^P]. The final concentrations of the proteins were 0, 0.1, 0.2, and 0.4 µM in lanes 1, 2, 3 and 4, respectively.

The effect of Rv2034 on its own expression was further examined by conducting a beta-galactosidase assay. A series of promoter-*lacZ* reporter plasmids was constructed using beta-galactosidase as reporter gene and transformed into *M. smegmatis*. Galactosidase activities in transformed cells were then measured. As shown in Figure 2A, the strain harboring pMZ+ that contains the strong promoter hsp60 had high beta-galactosidase activity (∼1600 Miller units) compared with the strain which harbors the pMZ0 plasmid containing the non-promoter lacZ plasmid (<100 Miller units), indicating that the reporter system works well. The promoter DNA of Rv2034 promoted the expression of lacZ (pMZ1), as a high level of beta-galactosidase activity was observed (∼2200 Miller units). Strikingly, when the Rv2034 regulator was introduced, the beta-galactosidase acitivity decreased sharply (Figure 2A, as indicated by pMZ2). This indicates that Rv2034 is capable of repressing the activity of its own promoter DNA and, consequently, its own expression.

### Identification of the Rv2034 residues essential for its DNA-binding activity

Our search for conserved domains [27] within the Rv2034 protein revealed two putative zinc binding sites at A32 and E35, an N-terminal dimerization interface and multiple putative DNA binding sites (Figure 2B, left panel, top bar). In addition, the C61 residue is likely critical for the formation of disulfide bonds. Mutations at C61 should therefore affect the dimerization of Rv2034. Three Rv2034 variants (Rv2034AE^A32G, E35G^, Rv2034ΔN28 with truncation of 28 N-terminal amino acid residues, and Rv2034C61A^C61A^; Figure 2B) were designed and their DNA-binding activities were assayed (see Materials and Methods section). The diagram shown in Figure 2B illustrates the differences between wild type and mutated proteins. As shown in Figure 2C, when 3 nM Rv2034 promoter DNA was co-incubated with increasing amounts of Rv2034 wild type and mutational proteins (0, 0.1, 0.2, 0.4, 0.8 µM), no shifted bands were observed for any of the three Rv2034 variants, indicating the all of the mutated proteins lacked the capacity to bind to the Rv2034 promoter DNA.

To further confirm this result, a series of promoter-lacZ reporter plasmids containing coding sequences of the mutated proteins were constructed and beta-galactosidase activity was measured in cells transformed with those plasmids. As shown in Figure 2A, strains expressing the Rv2034AE mutant protein had strikingly high beta-galactosidase activity (∼2200 Miller units), indicating that Rv2034AE failed to repress the active promoter, resulting in a high level of beta-galactosidase expression. Similarly, Rv2034 ΔN28 and Rv2034C61A also failed to repress the active promoter of Rv2034. These results indicate that the putative zinc binding sites, dimerization interface, and the only cysteine residue at C61 are all critical for the DNA-binding activity of Rv2034.

### Zinc and four other metal ions do not inhibit the DNA-binding ability of Rv2034

**Figure 3:**
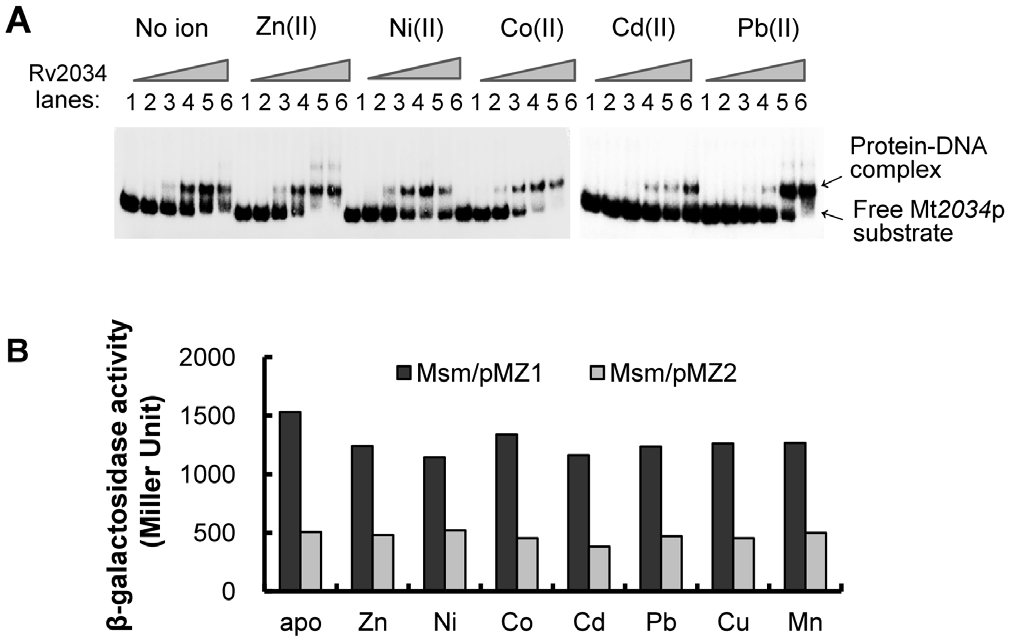
The effects of metal ions on the DNA-binding activity of Rv2034. (**A**) EMSA assays with or without metal ions. The Rv2034p DNA substrate was incubated with various amounts of the Rv2034 protein (0, 0.1, 0.2, 0.3, 0.4, and 0.5 µM) with or without 1 mM metal ions, as indicted on the top. (**B**) β-galactosidase activity was measured in *M. smegmatis* mc^2^ 155 containing the pMZ1 and pMZ2 reporter plasmids. Cells were cultured with no metal supplement or maximum permissive concentrations of Zn(II) (70 µM), Ni(II) (35 µM), Co(II) (15 µM), Cd(II) (75 µM), Pb(II) (10 µM), Cu(II) (50 µM), or Mn(II) (0.75 µM).

ArsR-type transcriptional regulators are known to function as metal sensors that sense and respond to metal ion stress [28]. Several lines of evidence show that the DNA binding activity of ArsR is inhibited by the presence of metal ion [12], [21]–[23]. Since our search for conserved domains [27] within the Rv2034 protein revealed two putative zinc binding sites, we further investigated the susceptibility of Rv2034 to several metal ions by conducting an EMSA assay. As shown in Figure 3A, when 1 mM metal ions of Zn(II) were co-incubated with Rv2034 promoter DNA and increasing amounts of Rv2034 (0, 0.1, 0.2, 0.4, 0.6, 0.8 µM), clear shifted bands were observed. This result indicated to us zinc ions are incapable of inhibiting the DNA-binding activity of Rv2034. Similarly, Ni(II), Co(II), Cd(II), and Pb(II) also failed to inhibit the activity of Rv2034, as shown in Figure 3A.

To further confirm this result, we performed beta-galactosidase assays. Strains harboring pMZ1 or pMZ2 reporter plasmids were cultured in minimal medium containing no excessive metal ions, as previously described [21], and beta-galactosidase activities in those strains were measured in the presence or absence of added metal ions. As shown in Figure 3B, the Msm/pMZ1 strains exhibited high level of beta-galactosidase activity (>1000 Miller units) while the Msm/pMZ2 strains showed low level of beta-galactosidase activity, irrespective of the presence of metal ions. Thus, all of the metal ions tested failed to alleviate Rv2034-mediated repression. Taken together, our results indicate that the DNA-binding ability of Rv2034 is not susceptible to Zn(II), Ni(II), Co(II), Cd(II), Pb(II), Cu(II), and Mn(II) metal ions.

### Rv2034 forms dimer *in vitro*

**Figure 4:**
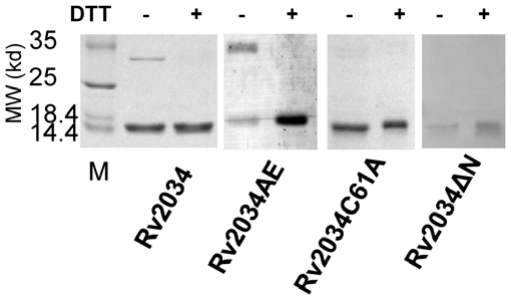
Rv2034 forms dimer *in vitro*. Rv2034 wild type and mutant proteins were boiled in the sample buffer in the absence or presence of 10 mM dithiothreitol (DTT) as indicated on the top. The protein size markers are labeled on the left.

Several transcriptional regulators, such as CmtR [23], are known to exist as dimer or multimer. The purified protein of Rv2034 always forms an insoluble precipitate under low salt ion conditions (data not shown), implying that Rv2034 may also form dimers or multimers. Polyacrylamide gel electrophoresis (PAGE) under non-reducing or reducing conditions was performed to examine the dimerization status of Rv2034. As shown in Figure 4, in the absence of the reducing agent 0.1 M dithiothreitol (DTT), two bands of approximately 15 kDa and 30 kDa, which correspond to Rv2034 monomer and dimer, respectively, were observed. By contrast, when 0.1 M DTT was added, only one band that was stronger than the corresponding band with DTT was observed, as shown in Figure 4. This result suggested that Rv2034 can form dimer *in vitro*. Interestingly, we found that the Rv2034AE mutant protein retained the capacity to form dimers while Rv2034ΔN and Rv2034C61A didn't (Figure 4).

### Rv2034 binds with DNA fragments containing a palindrome sequence motif

**Figure 5:**
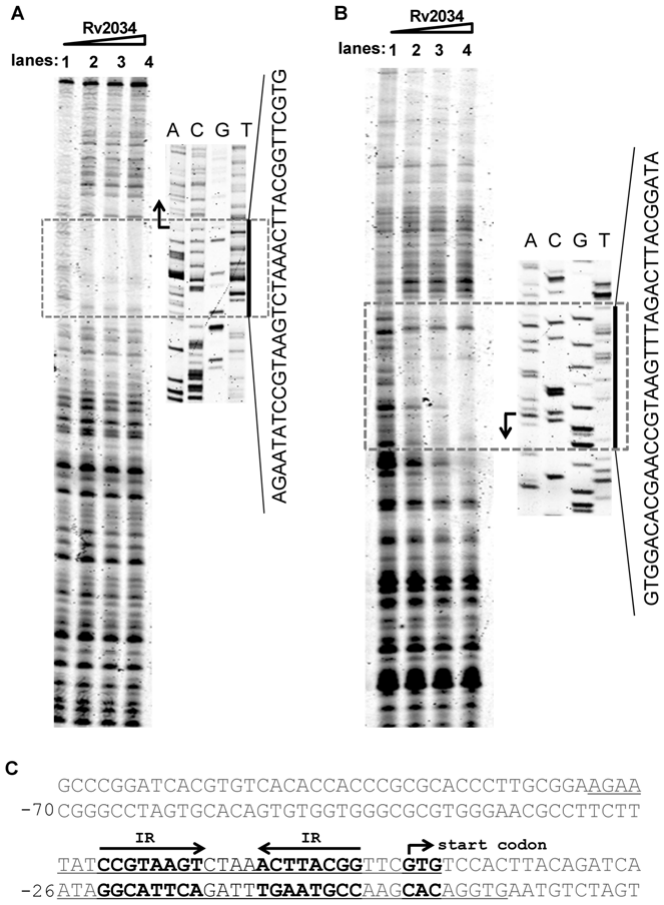
Characterization of the DNA binding sites of Rv2034. DNaseI footprinting assays of the coding strand (**A**) and the non-coding strand (**B**). The ability of Rv2034 to protect the Rv2034 promoter DNA against DNaseI digestion was investigated in the presence of increasing amounts of Rv2034 (lanes 1–4). The sequencing ladders are shown on the side and the corresponding protected regions are indicated by black bars. The DNA sequence of protected regions were read from the sequencing ladder and shown. (**C**) Sequence and structural characteristics of the protected Rv2034 promoter regions. The regions protected are underlined and the 20 bp sequences containing the inverted repeats (IR) are shown in bold. The translation start codon of Rv2034 is also indicated in bold.

The Rv2034 binding motif in its promoter DNA was characterized by DNaseI footprinting assays. As shown in Figure 5A, when increasing amounts of the Rv2034 protein (0–1 µM) were co-incubated with its DNA substrate and DNaseI, the region around AGAATATCC GTAAGTCTAAACTTACGGTTCGTG was clearly protected on the coding strand. This result indicated that this DNA fragment contained a potential binding motif for Rv2034. Similarly, the region around CACGAACCGTAAGTTTAGACTTACGGATA was protected when the non-coding strand DNA was used as substrate (Figure 5B). The protected DNA region extended from position −30 to +3 in the coding strand, and from position −26 to +8 in the non-coding strand (Figure 5C). In both of those protected regions, a palindromic motif formed by two inverted repeats (IR, 5′-CCGTAAGT-3′) that were separated by four nucleotides (5′-CTAA-3′ or 5′-TTAG-3′) was found.

**Figure 6:**
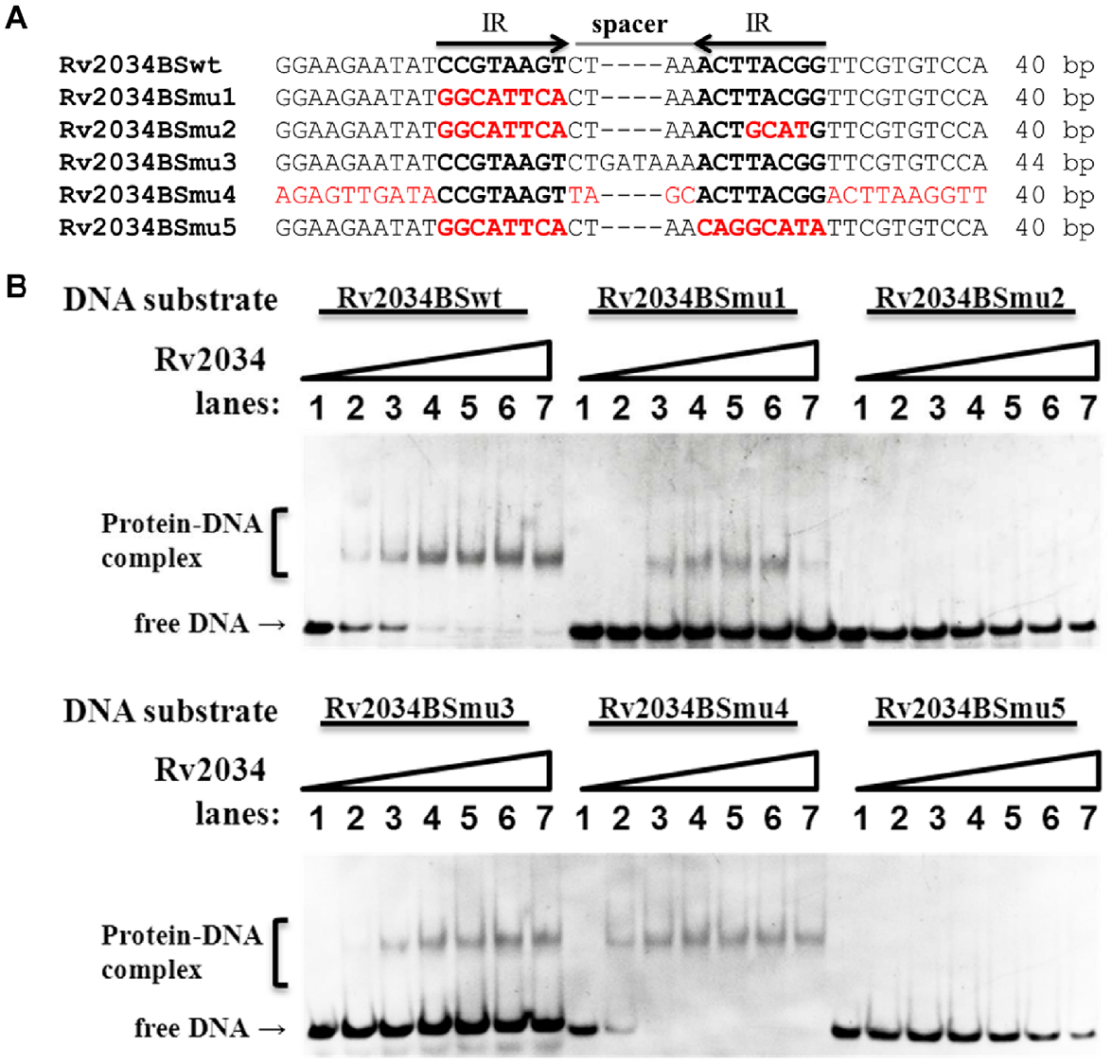
EMSA assays for the ability of Rv2034 to bind variant DNA substrates. (**A**) A diagram showing the DNA substrates used in this assay. The DNA substrates are 40 bp or 44 bp long, and the binding sites in them are shown in bold. The inverted repeats (IR) and the “spacer” sequence between inverted repeats are indicated on the top. Sequences are aligned and the mutated region(s) are indicated in red. (**B**) EMSA assays for the DNA-binding activity of Rv2034 on the DNA substrates listed in (A). Each DNA substrate was co-incubated with 0, 0.1, 0.2, 0.4, 0.8, 1 and 1.2 µM of Rv2034 proteins (final concentration for lanes 1–7, respectively).

We then performed EMSA assays to confirm the significance of the motif for specific recognition by Rv2034. As shown in Figure 6A, a 40 bp DNA fragment derived from the promoter DNA of Rv2034 and its five mutated variants (Rv2034BSmu1, Rv2034BSmu2, Rv2034BSmu3, Rv2034BSmu4, and Rv2034BSmu5) were used as substrates in the EMSA assay. The differences between wild type and mutated DNA fragments are indicated in red and the inverted repeats and binding sites are in bold, as indicated by arrows in Figure 6A. The Rv2034BSmu1 DNA fragment retained the right side of the palindromic sequence or a single binding site. In Rv2034BSmu2, both of the inverted repeats were replaced by random sequences (Figure 6A). In the substrate Rv2034BSmu3, the length of the spacer DNA between the two inverted repeats was doubled from “CTAA” to “CTGATAAA” (Figure 6A). In Rv2034BSmu4, all but the inverted repeats were replaced by random sequences (Figure 6A). In Rv2034BSmu5, the entire inverted repeats were replaced by random sequences (Figure 6A). Our EMSA results indicated that Rv2034 was able to bind with the DNA substrates Rv2034BSwt, Rv2034BSmu1, Rv2034BSmu3 and Rv2034BSmu4, but not with Rv2034BSmu2 and Rv2034BSmu5, as shown in Figure 6B. These data indicate that a single binding site or half of the palindromic sequence is sufficient for Rv2034 binding. Notably, binding with DNA fragments that had both the binding sites (and the palindrome) and/or an ideal spacer length (4 bp) was observed to be stronger than those with a single binding site, as indicated by less free DNA in the former case (Figure 6B). In summary, our results indicate that the binding site for Rv2034 contains a specific palindromic sequence motif.

### Rv2034 is a functional regulator of the *dosR* gene in *M. tuberculosis*

**Figure 7:**
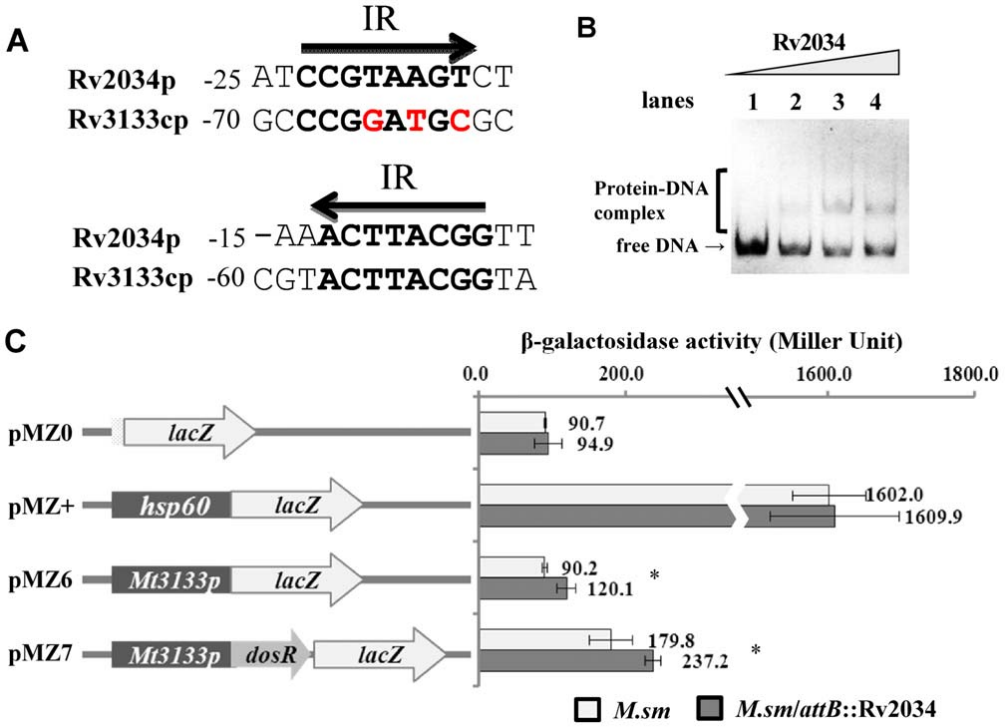
Regulation of the *dosR* gene by Rv2034. (**A**) Alignment of the promoter DNAs of Rv2034p and Rv3133cp. The numbers indicate the distance from the start codon of each gene. The inverted repeats (IR) are shown in bold and mismatches are highlighted in red. (**B**) EMSA assays for the DNA-binding activity of Rv2034 on the promoter DNA of Rv3133cp. The DNA substrate was co-incubated with 0, 0.2, 0.4, and 0.8 µM of Rv2034 proteins. (**C**) The effect of Rv2034 on the *dosR* promoter region was assayed by constructing a series of promoter-lacZ fusion plasmids. Null promoter-lacZ (pMZ0) and hsp60-lacZ (pMZ+) were used as controls. The names of lacZ-fused plasmids are listed in the left panel, and the elements differing among them are shown in the middle panel. Right panel: The activity of β-galactosidase for the corresponding reporter plasmids were measured in the *M.sm* wild type strains (hollow bar) or the *M.sm*/*attB*::Rv2034 strains (solid bar). β-galactosidase activities (Miller units) are reported as averages from three independent experiments. Errors bars represent standard deviations. For statistical analyses, independent two-sample student's t-tests were performed. Significant differences (P<0.05) are indicated by asterisks (*).

Characterization of the binding motif of Rv2034 made it possible for us to search putative target genes regulated by the Rv2034 protein. Putative promoter regions (upstream 250 bp) of entire open reading frames were searched in the *M. tuberculosis* genome for putative Rv2034 binding motifs. Other than the promoter of Rv2034 (or Rv2033c), five promoters (Rv1823, Rv1997, Rv2621c, Rv2622, and Rv3133c) were found to contain the potential Rv2034 binding site, as shown in Figure S1. Remarkably, the promoter DNA of Rv3133c has an inverted repeat with exact match to the Rv2034 binding motif we identified, indicating that Rv3133c could be regulated by Rv2034 through a single binding site, as shown in Figure 7A. This led us to hypothesize that Rv2034 could be a potential regulator of the DosR protein encoded by Rv3133c. To test this prediction, the promoter DNA of Rv3133c was amplified and an EMSA assay was conducted. As shown in Figure 7B, when 3 nM *dosR* promoter DNA was co-incubated with increasing amounts of Rv2034 (0, 0.2, 0.4, 0.8 µM), clear shifted bands were observed.

We then performed beta-galactosidase reporter assays. We first created an integrated strain in which a mycobacteria-*E. coli* shuttle vector pMV361 containing Rv2034 coding sequence was integrated in the bacterial genome at an *attB* site. The pMZ6 plasmid, in which the *dosR* promoter sequence was placed upstream of *lacZ*, and the pMZ7 plasmid, in which the *dosR* promoter-*dosR* coding sequence was cloned upstream of *lacZ*, were transformed into the wild type strain (M.sm) and the integrated strain (M.sm/attB::Rv2034), respectively, to create recombinant strains. In the latter, Rv2034 was expressed and functioned as a regulator for the *dosR* promoter in *trans*. As shown in Figure 7C, the beta-galactosidase acitivities for pMZ0 or pMZ+ showed no significant differences between the wild type strain and the integrated recombinant strains. However, the integrating strains with the pMZ6 vector were observed to have ∼30% higher beta-galactosidase activity (∼120 Miller units) than the wild type (∼90 Miller units). Similarly, the integrating strains with the pMZ7 vector also showed higher beta-galactosidase activity (∼237.2 Miller Units) compared with the wild type (179.8 Miller Units). These results show that Rv2034 binds with the promoter DNA of the *dosR* gene and promotes gene expression solely or in association with DosR. Taken together, our results indicate that Rv2034 is a functional regulator of the *dosR* gene in *M. tuberculosis*.

## Discussion

DosR is the primary mediator of hypoxic response [3], [6], and is required for host-pathogen interaction in *M. tuberculosis* [29]. However, the regulatory mechanism of DosR remains unclear. In this study, we characterized an ArsR transcriptional regulator, encoded by Rv2034 in *M. tuberculosis*, as the regulator of DosR. We demonstrated that Rv2034 is capable of binding with the promoter DNA of DosR and positively regulating DosR expression. To the best of our knowledge, this is the first report on the characterization of a regulator of the *dosR* gene.

ArsR-type transcriptional regulators are known to act as metal sensors that mediate response to metal ion stress [28]. Binding of metal ions to ArsR proteins lead to allosteric effects that switch on or off the protein's DNA-binding capability [30]. By utilizing the Conserved Domains Database [27], we found that Rv2034 has two putative zinc binding sites. This led us to first examine whether it senses and responds to zinc ion like the other ArsR homology protein SmtB (encoded by Rv2358) does [24]. However, our results revealed that zinc ions, as well as six other metal ions, are not able to inhibit the DNA-binding activity of Rv2034 or to alleviate Rv2034-mediated repression. Although we cannot rule out the possibility that Rv2034 can sense metal ions that we did not test, our results strongly suggest that Rv2034 responds to signals other than metal ions.

Our experiments revealed that Rv2034 acts as an auto repressor and, interestingly, positively regulates the expression of *dosR*, *phoP*, and *groEL2* [10]. Differences in the distances between Rv2034 binding sites and the start codon may underlie such different effects of Rv2034 binding. The binding site of Rv2034 within its own promoter DNA is much closer to its coding sequence compared with that of *phoP* and *groEL2*. It is likely that the complex formed by Rv2034 binding with its promoter DNA inhibits the binding or sliding of RNA polymerase, causing auto-repression. On the contrary, binding at a further distance may be conducive to initiation or promotion of transcription. This kind of dual effects of transcriptional regulators was also observed in other report [31].

It is noteworthy that the binding activity of Rv2034 to its own promoter is much stronger than to the promoter of other genes (i.e. *phoP*). In the EMSA assays, the amount of Rv2034 protein needed to sufficiently bind with the promoter of Rv2034 is about 0.2 µM, while 2 µM of the protein was needed to bind with the promoter of *phoP* or *groEL2* [10]. In addition, our mutational analysis (Figure 6) of the DNA motif that Rv2034 binds to revealed that a single binding motif is sufficient for Rv2034 binding. The results indicated that affinity is greatly increased when both sites are present, implying the presence of a cooperative binding effect, similar to some of the TetR family regulators [32]. Several other studies have identified many Rv2034 target genes [10], [25], [33], [34]. Using a systematic ChIP-seq approach, the James Galagan group identified 614 unique binding sequences (representing 821 putative unique target genes, more than a quarter of all the genes in the genome) for Rv2034 in two separated experiments (available free in TBDB). These findings suggest that Rv2034 may act as a functional “global” regulator in *M. tuberculosis*. So the binding of Rv2034 has a certain extent of flexibilities. Among all the promoters, binding to its own promoter at 5′-CCGTAAGT∼N{4}∼ACTTACGG-3′ should be the strongest.

Several lines of evidence show that Rv2034-Rv2036 acts as one functional operon in *M. tuberculosis*. The coding sequences of these genes are not separate but overlapped in the *M. tuberculosis* genome. The Pearson correlation coefficient values of Rv2034/Rv2035, Rv2034/Rv2036, and Rv2035/Rv2036 (0.85, 0.72 and 0.70, respectively, as provided by TBDB [33], [34]; see supplementary Figure S2) also imply that they are co-expressed in many different conditions. In other words, the capability of auto regulation in Rv2034 should also affect the expression of Rv2035. Sequence analysis revealed that Rv2035 contains a polyketide_cyc2 domain, which is found in polyketide cyclase/dehydrase and lipid transport, implying a potential role of Rv2034 in the regulation of lipid metabolism related genes. Furthermore, analysis of large-scale DNA MicroArray datasets has shown that Rv2034 expression is significantly upregulated in media containing palmitic acid [34], which is a saturated fatty acid and the major fat component of dairy products. Notably, a remarkable number of putative Rv2034 target genes (such as *fadE5, 26, 28*, and *desA2*) are involved in lipid metabolism (Table 1). Moreover, the response regulator, PhoP, which regulates expression of the *pks2* and the *msl3* gene clusters [35] and accounts for sulfolipid, diacyltrehalose and polyacyltrehalose synthesis in *M. tuberculosis* H37Rv [36], has also been shown to be regulated by Rv2034 [10]. Taken together, these findings strongly suggest that Rv2034 may play an important role in the regulation of lipid metabolism.

**Table 1:** The collection of genes regulated by Rv2034.

*Rv#*	*Gene*	*Methods (refs)*	*Putative function (refs)*
Rv0244	*fadE5*	B1H [25]	EHR [8], Lipid metabolism
Rv0350	*dnak*	B1H [25]	EHR [8]
Rv0440	*groEL2*	B1H [25], ChIP-seq [33], [34]	
Rv0757*	*phoP*	B1H [25], EMSA [10], ChIP [10]	Lipid metabolism
Rv1094	*desA2*	B1H [25], ChIP-seq [33], [34]	Lipid metabolism
Rv1909c*	*furA*	B1H [25], ChIP-seq [33], [34]	EHR [8]
Rv2034*		B1H, EMSA,	EHR [8]
Rv2031c	*hspX*	B1H [25], SPR [25]	IHR [8]
Rv3133c*	*dosR*	EMSA, ChIP-seq [33], [34]	IHR [8]
Rv3197A*	*whiB7*	B1H [25]	EHR [8]
Rv3504	*fadE26*	B1H [25]	Lipid metabolism
Rv3545c	*fadE28*	B1H [25], ChIP-seq [33], [34]	EHR [8], Lipid metabolism

These genes have been characterized by one or more methods (B1H, EMSA, ChIP, ChIP-seq, etc.). Some of them were critical transcriptional regulators.

* indicates by which encoded gene is a transcriptional regulator. Abbreviates: B1H, bacterial one-hybrid assay; EMSA, electrophoretic mobility shift assay; ChIP, Chromatin immunize precipitation assay; ChIP-seq, Chromatin immunize precipitation following DNA sequencing; IHR, initial hypoxic response; EHR, enduring hypoxic response;

Eight Rv2034 target genes have been found to be differentially expressed in hypoxic conditions (Table 1). Interestingly, six genes among them are enduring hypoxic response genes, while two are initial hypoxic response genes [8]. Expression of the enduring hypoxic response genes were significantly induced at four and seven days of hypoxia, while expression of the initial hypoxic response genes which belong to the *dosR* regulon were transiently induced during the beginning phase of hypoxia [8]. To our knowledge, Rv2034 is the first regulator known to be involved in both of the two kinds of hypoxic responses. Interestingly, Rv2034 regulates *dosR*, *furA* and *whiB7*. Such interactions among different regulators can help enrich the complexity of the regulatory network and benefit the adaptation of the cell to hypoxic conditions. In addition, it is possible that Rv2034 is involved in the transition of hypoxic responses. Besides, in the ArsR knock-out strain of M. smegmatis, in which the coding region of Rv2034 homologous gene (encoded by Ms6762) was replaced by the hygromycin gene, is more resistant to hydrogen peroxide (H2O2), as compared with the wild type and complementary strains (see supplementary figure S3). This implies that the homologous gene of Rv2034 play a role in the adaptation of oxidative stress in *M. smegmatis* and shed a light on the possible role of Rv2034 in *M. tuberculosis* H37Rv. A comprehensive understanding of the function of Rv2034 should be uncovered along with more advances in system biology.

In summary, we characterized the auto repression of the ArsR transcriptional regulator Rv2034 and identified a palindromic sequence as its binding site. Several residues in Rv2034 critical to its DNA binding activities were successfully characterized. Notably, we found that Rv2034 regulates the expression of the *dosR* gene. Our findings establish a direct link of the ArsR regulator to both lipid metabolism and hypoxic adaptation in *M. tuberculosis*.

## Materials and Methods

### Plasmids, strains, enzymes and chemicals

The *Escherichia coli* BL21 cells and the pET28a vector were purchased from Novagen. All the enzymes including restriction enzymes, ligase, and DNA polymerase were purchased from TaKaRa Biotech. Deoxynucleoside triphosphates (dNTPs) and all antibiotics were purchased from TaKaRa Biotech as well. PCR primers were synthesized by Invitrogen (See Table S1). All the derived plasmids were listed in Table S2. Ni-NTA (Ni^2+^-nitrilotriacetate) agarose was obtained from Qiagen.

### Cloning, expression, and purification of mycobacterial proteins

Protein expression and purification were performed as previously described [10]. Rv2034AE^A32G, E35G^ and Rv2034C61A^C61A^ mutated variants were generated by site-directed mutagenesis using the overlap extension polymerase chain reaction technique (overlapping-PCR). Primers used in the overlapping-PCR are listed in supplementary Table S1. The Rv2034ΔN mutated variant was generated by using a truncated forward primer Rv2034ΔNf-EcoRI and the primer Mt2034pr-XbaI for PCR (see Table S1).

### Electrophoretic mobility shift assay

Large DNA segments with more than 70 base pairs were acquired using polymerase chain reaction (PCR), and short DNA segments were synthesized and directly annealed *in vitro*. The primers used for PCR are listed in Supplemental Table S1. All the DNA substrates used in this assay are listed in Table S3. DNA fragments used in this assay were labeled at the 5′-end with [γ-^32^P] ATP or with fluorescein isothiocyanate (FITC). Mixtures containing the labeled DNA (∼10 nM) and increasing concentrations of the protein (as indicated in the legend of the corresponding figure) were incubated in EMSA buffer (100 mM Tris-HCl, pH 8.0, 100 mM NaCl, 1 mM DTT and 10% glycerol) for 30 min at room temperature, then subjected to 5% native polyacrylamide gel electrophoresis (PAGE). Electrophoresis was performed at 150 V at room temperature for about 1.5 hours in 0.5× Tris-borate-EDTA buffer (TBE). Radioactive gels were exposed to a storage-phosphor screen (GE healthcare) overnight at room temperature. Images of gels were acquired using a Typhoon Scanner (GE healthcare).

### Bacterial one-hybrid assay

Bacterial one-hybrid assays were performed as previously described [25]. In the present study, the promoter region of Rv2034 and other reference DNA fragments were amplified using corresponding primer pairs (see Table S1) and cloned upstream of the reporter genes (*HIS3* and *aadA*) in the pBXcmT plasmid [25]. In this way, a series of reporter plasmids (see Table S2) were created. The reporter plasmids were co-transformed with the pTRG-Rv2034 plasmid (see Table S2) into *Escherichia coli* XR competent cells. Co-transformants were grown on selective medium containing 20 mM 3-amino-1, 2, 4-triazole (3-AT), 16 µg/mL streptomycin, 15 µg/mL tetracycline, 34 µg/mL chloramphenicol, and 50 µg/mL kanamycin. Plates were incubated at 30 degree centigrade for 3–4 days. Co-transformants containing the pBX-MthspXp and pTRG-Rv3133c plasmids [25] served as the positive control while co-transformants containing the pBX-MthspXp and pTRG-Rv3133cΔC plasmids [25] served as the negative control. Co-transformants containing the empty vectors pBX and/or pTRG were used as self-activation controls.

### DnaseI footprinting assay

The substrate DNAs used in this assay were about 220 base pairs long, including 140 base pairs upstream from the start codon and the first 82 base pairs of the Rv2034 coding region. Both the coding and non-coding strands were amplified by PCR using their specific primers labeled with Fluorescein Isothiocyanate (FITC) (see Table S3). PCR products were purified with BioFlux PCR DNA Purification kit (BioFlux) and subjected to the same binding reaction as in EMSA. DNaseI footprinting was performed as previously described [37]. The ladders were produced using the Sanger dideoxy method.

### β-galactosidase activity assay

β-galactosidase activity assays were performed as previously described [37]. In the present study, the experiments were performed by creating operon-lacZ fusions based on the expression vector pMV261 [38]. To obtain the plasmid pMZ1 to pMZ9, a series of digestion and ligation reactions were carried out. The promoter sequences, the corresponding regulatory protein coding sequences and the reporter gene *lacZ* were cloned into the pMV261 backbone step by step (See Table S2). Taking the construction of the pMZ3 plasmid as an example, the putative promoter region of Rv2034 Mt2034p was first cloned into the pMV261 backbone using *Xba*I/*EcoR*I. Then, the coding sequence of Rv2034AE was cloned using *Eco*R/*Hind*III. Finally, the reporter gene *lacZ* was cloned using *Hind*III/*Nhe*I. To construct the pMZ+ plasmid, which was used as a positive control in our assays, the reporter gene *lacZ* was inserted into pMV261 downstream of the enhanced promoter of *hsp60* using *Hind*III/*Nhe*I. To construct the negative control plasmid pMZ-, the promoter region of pMZ+ was removed by *Xba*I/*EcoR*I double-digest and blunt-end ligation. The reporter plasmids were transformed into the *M. smegmatis* str. mc^2^ 155 host strain and the corresponding recombinant reporter strains were obtained. All strains were grown at 37°C to an OD_600_ of ∼1.5. Cell suspensions (1 ml) were then collected and washed with cold PBS buffer (137 mM NaCl, 2.7 mM KCl, 8 mM Na_2_HPO4, 1.46 mM KH_2_PO_4_, pH 7.4) once or twice. For cell permeabilization, 600 µl of the Z buffer (60 mM Na_2_HPO_4_, 40 mM NaH_2_PO_4_, 10 mM KCl, 1 mM MgSO_4_ and 59 mM β-mercaptoethanol, pH 7.5) was dispensed into the microcentrifuge tube to resuspend the cells. Three hundred microliters of resuspended cells was saved to determine the A_600_ value. After adding 50 µl of 0.1% SDS (sodium dodecyl sulfonate) and 100 µl of chloroform, the mixture was vortexed and incubated at 28°C for 5 min. A total of 200 µl of ONPG (o-nitrophenyl-D-galactoside, 4 mg/ml) was then added to a final concentration of 0.66 mg/ml. Each tube was incubated at room temperature for 5–10 min. Five hundred microliters of 1 M Na_2_CO_3_ was added to end this reaction. Each tube was centrifuged at 1200 rpm for 5 min and the supernatant was used to determine the A_420_ and A_550_ values. β-galactosidase activities were calculated as described previously [39]. This system is similar to the pJEM system [40], [41] and works well in terms of repeatability, distinction and reproducibility as according to the practical operations [10], [37].

## Supporting Information

Figure S1 **Search for putative target genes regulated by the Rv2034 protein using the binding motif of Rv2034.** Putative promoter regions (upstream 250 bp) of entire open reading frames in the *M. tuberculosis* genome were searched based on the Rv2034 binding motif (or the palindrome sequence) that we identified. Other than the promoter of Rv2034 (or Rv2033c), we found that five promoters (Rv1823, Rv1997, Rv2621c, Rv2622, and Rv3133c) contained the potential Rv2034 binding site. The promoter regions were aligned and mismatches are shown on the right.
(TIF)

Figure S2 **Pearson correlation coefficient values of Rv2034/Rv2035, Rv2034/Rv2036, and Rv2035/Rv2036 pairs.** Correlation coefficient values were obtained from TBDB. Co-expressed genes are likely to have higher coefficients than ones that are not co-expressed (from 0 to 1).
(TIF)

Figure S3 **Construction of an ArsR-knockout strain and the growth determination in the presence and absence of hydrogen peroxide (H_2_O_2_).** (**A**) Construction of the Ms6762 knockout strain of *M. smegmatis* and Southern blot assays. Ms6762 is the homologous gene of Rv2034 in *M. smegmatis*. Left panel: Using a recombination-based strategy, the coding sequence of Ms6762 was replaced by a hygromycin resistance gene (indicated as hyg in the schematic representation). The restriction sites for NarI are indicated with arrows. The DNA probes used in Southern blotting are indicated with black bars. Right panel: Southern blot assays. The DNA fragment corresponding to the region upstream of Ms6762 in *M. smegmatis* was obtained by PCR and labeled with digoxigenin dUTP (Boehringer Mannheim, Inc., Germany). The probe was used to detect the change in size of the NarI-digested genomic fragment of *M. smegmatis* wild type (WT, ∼600 bp) and knock-out (KO, ∼900 bp) strains. The knock-out, the wild type and the complement strains were cultured in the presence of 0 mM (B), 15 mM (C) and 20 mM (D) of hydrogen peroxide, as indicated on the top. The complement strain was generated by transforming an inducible expression vector of pMind-Ms6762 into the verified Ms6762 knock-out strain. To induce the expression of Ms6762 in the complement strains, ∼25 ng/ml of the inducer (tetracycline) was added in the corresponding culture. The experiments were performed thrice with similar results.
(TIF)

Table S1 **Primers used in this study.**
(DOC)

Table S2 **Plasmids used in this study.**
(DOC)

Table S3 **DNA substrates used in this study.**
(DOC)
