# Supplementary figures and images for: Characterization of a Novel ArsR-Like Regulator Encoded by Rv2034 in Mycobacterium tuberculosis
Source: PLoS One. 2012 Apr 27;7(4):e36255. doi: 10.1371/journal.pone.0036255 (PMC3338718; doi:10.1371/journal.pone.0036255)

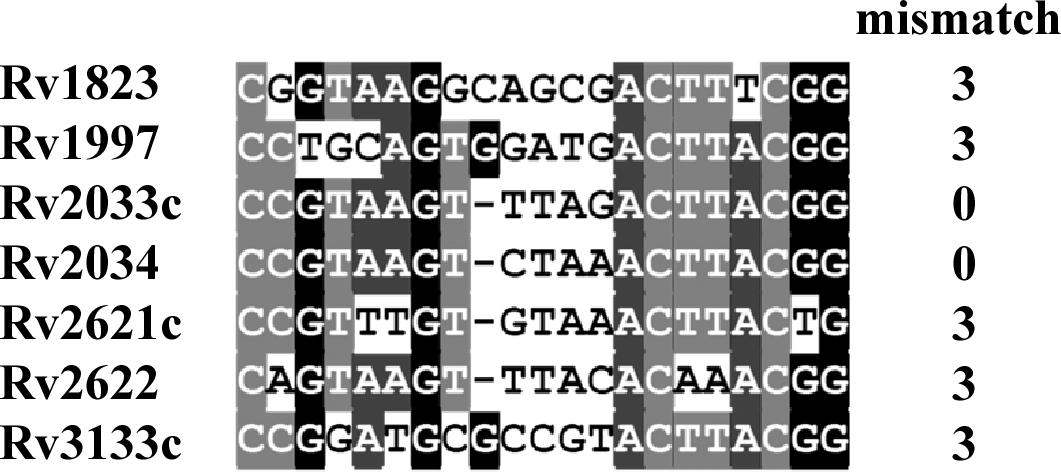

Supplement: Figure S1 — Search for putative target genes regulated by the Rv2034 protein using the binding motif of Rv2034. Putative promoter regions (upstream 250 bp) of entire open reading frames in the M. tuberculosis genome were searched based on the Rv2034 binding motif (or the palindrome sequence) that we identified. Other than the promoter of Rv2034 (or Rv2033c), we found that five promoters (Rv1823, Rv1997, Rv2621c, Rv2622, and Rv3133c) contained the potential Rv2034 binding site. The promoter regions were aligned and mismatches are shown on the right. (TIF) [file pone.0036255.s001.tif]

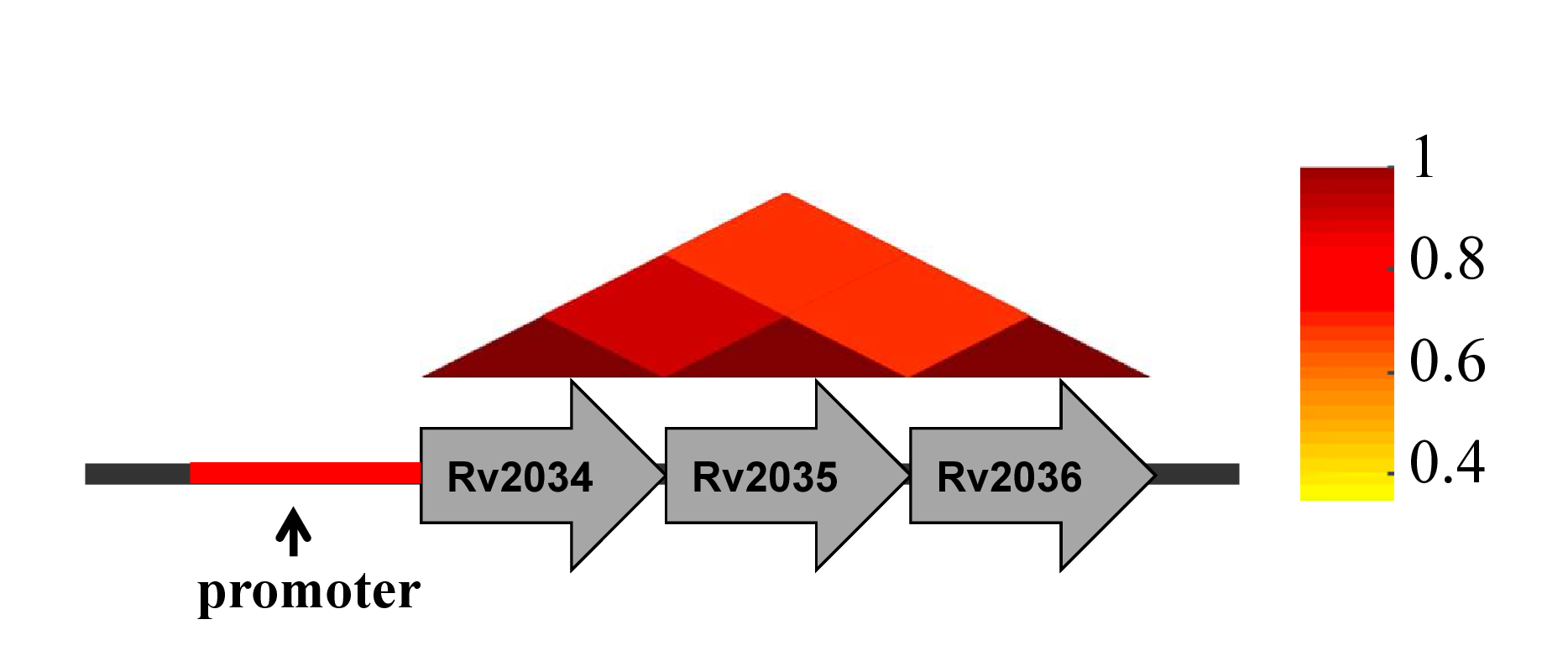

Supplement: Figure S2 — Pearson correlation coefficient values of Rv2034/Rv2035, Rv2034/Rv2036, and Rv2035/Rv2036 pairs. Correlation coefficient values were obtained from TBDB. Co-expressed genes are likely to have higher coefficients than ones that are not co-expressed (from 0 to 1). (TIF) [file pone.0036255.s002.tif]

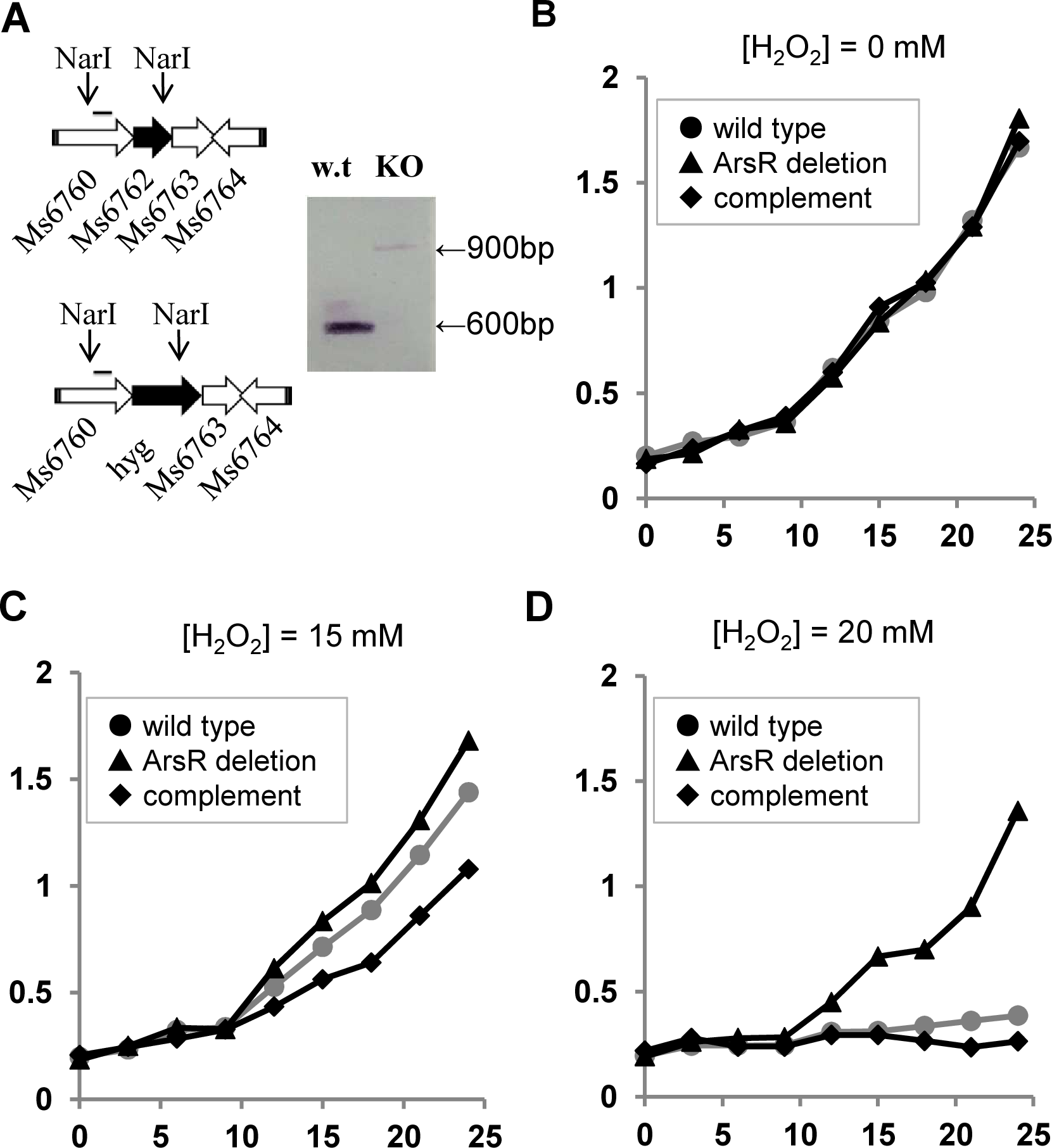

Supplement: Figure S3 — Construction of an ArsR-knockout strain and the growth determination in the presence and absence of hydrogen peroxide (H2O2). (A) Construction of the Ms6762 knockout strain of M. smegmatis and Southern blot assays. Ms6762 is the homologous gene of Rv2034 in M. smegmatis. Left panel: Using a recombination-based strategy, the coding sequence of Ms6762 was replaced by a hygromycin resistance gene (indicated as hyg in the schematic representation). The restriction sites for NarI are indicated with arrows. The DNA probes used in Southern blotting are indicated with black bars. Right panel: Southern blot assays. The DNA fragment corresponding to the region upstream of Ms6762 in M. smegmatis was obtained by PCR and labeled with digoxigenin dUTP (Boehringer Mannheim, Inc., Germany). The probe was used to detect the change in size of the NarI-digested genomic fragment of M. smegmatis wild type (WT, ∼600 bp) and knock-out (KO, ∼900 bp) strains. The knock-out, the wild type and the complement strains were cultured in the presence of 0 mM (B), 15 mM (C) and 20 mM (D) of hydrogen peroxide, as indicated on the top. The complement strain was generated by transforming an inducible expression vector of pMind-Ms6762 into the verified Ms6762 knock-out strain. To induce the expression of Ms6762 in the complement strains, ∼25 ng/ml of the inducer (tetracycline) was added in the corresponding culture. The experiments were performed thrice with similar results. (TIF) [file pone.0036255.s003.tif]
