# Supplementary material for: Characterization of a Novel ArsR-Like Regulator Encoded by Rv2034 in Mycobacterium tuberculosis
Source: PLoS One. 2012 Apr 27;7(4):e36255. doi: 10.1371/journal.pone.0036255 (PMC3338718; doi:10.1371/journal.pone.0036255)
Supplement: Table S1 — Primers used in this study. (DOC) [file pone.0036255.s004.doc]

## Supplemental Table 1

Primers used in this study

| **Primer Name** | **Sequence (5’-3’)** | **Restriction site** | **Usage** |
| --- | --- | --- | --- |
| Mt2034pf-EcoRI | AATTGAATTCGCCTGCGGCAAGCACGTCGG | EcoRI | Clone and expression |
| Mt2034pr-XbaI | CCGGTCTAGAGAACCGTAAGTTTAGACTTA | XbaI | Clone and expression |
| Rv2034AE-f | ATCGTGGAGCGGCTGGCGCACGGCCCGCTGGGCGTCGGCGGGTTGGCCCGCGACCTGCCCGTCAGCCGAC |  | overlapping PCR |
| Rv2034AE-r | GTCGGCTGACGGGCAGGTCGCGGGCCAACCCGCCGACGCCCAGCGGGCCGTGCGCCAGCCGCTCCACGAT |  | overlapping PCR |
| Rv2034C61Af | CTCAAGACCGCCAGGCTGGTGGCGGACCGCCCCGCGGGAACACGC |  | overlapping PCR |
| Rv2034C61Ar | GCGTGTTCCCGCGGGGCGGTCCGCCACCAGCCTGGCGGTCTTGAG |  | overlapping PCR |
| Rv2034ΔNf-EcoRI | ATATGAATTCTAGGCCCGCTGGCCGTCGGCGA | EcoRI | Clone and expression |
| lacZf-Hind3 | ATTCAAGCTTATGAGGATGAGGGAAGCAAG | HindIII | lacZ-fusion |
| lacZr-NheI | ATGCGCTAGCTTATTTTTGACACCAGACCA | NheI | lacZ-fusion |
| Mt2034pf-XbaI | AATTTCTAGAGCCTGCGGCAAGCACGTCGG | XbaI | lacZ-fusion, bacterial one-hybrid assy |
| Mt2034pr-EcoRI | CCGGGAATTCGAACCGTAAGTTTAGACTTA | EcoRI | lacZ-fusion, bacterial one-hybrid assy |
| Mt440pf-XbaI | AATTTCTAGACGAACGAGGGGCATGACCCG | XbaI | lacZ-fusion, bacterial one-hybrid assy |
| Mt440pr-EcoRI | GACCGAATTCTTGCGAAGTGATTCCTCCGG | EcoRI | lacZ-fusion, bacterial one-hybrid assy |
| Rv2034r-Hind3 | ATGCAAGCTTTCATGTGTCGTCTCCTTCGG | HindIII | lacZ-fusion |
| Mt3133cpf-XbaI | ACTGTCTAGAGTTGGCGCATGTACACCTGAGCCGT | XbaI | lacZ-fusion |
| Mt3133cpr-EcoRI | AGATGAATTCCAGGGCACCACTCCCAAGATCCGCT | EcoRI | lacZ-fusion |
| Mt3133cr-EcoRI | ATATGAATTCTCATGGTCCATCACCGGGTGGCCGC | EcoRI | lacZ-fusion |
