# Supplementary material for: Characterization of a Novel ArsR-Like Regulator Encoded by Rv2034 in Mycobacterium tuberculosis
Source: PLoS One. 2012 Apr 27;7(4):e36255. doi: 10.1371/journal.pone.0036255 (PMC3338718; doi:10.1371/journal.pone.0036255)
Supplement: Table S2 — Plasmids used in this study. (DOC) [file pone.0036255.s005.doc]

## Supplemental Table 2

Plasmids used in this study

| **Plasmid Name** | **Features** | **Source or refers** |
| --- | --- | --- |
| pBT | chlo^r^, p15A replicon, lac-UV5 promoter | Stratagene |
| pBXcmT | pBT derivative used in B1H assay | [1] |
| pBX-MthspXp | pBX derivative containing MthspXp | [1,2] |
| pBX-MtgroEL2p | pBX derivative containing MtgroEL2p | This study |
| pBX-Mt2034p | pBX derivative containing Mt2034p | This study |
| pTRG | tet^r^, ColE1 replicon, lpp/lac-UV5 promoter | Stratagene |
| pTRG-Rv2034 | Rv2034 in EcoRI-XbaI sites of pTRG | This study |
| pTRG-Rv3133c | Rv3133c in EcoRI-XbaI sites of pTRG | [1] |
| pTRG-Rv3133cΔC | Rv3133cΔC in EcoRI-XbaI sites of pTRG | [1] |
| pET28a(+) | Kan^r^, T7 lac promoter, N-terminal His_6_ | Novagen |
| pET-Rv2034 | Rv2034 in EcoRI-XbaI sites of pET28a | This study |
| pET-Rv2034AE | Rv2034AE in EcoRI-XbaI sites of pET28a | This study |
| pET-Rv2034C61A | Rv2034C61A in EcoRI-XbaI sites of pET28a | This study |
| pET-Rv2034ΔN | Rv2034ΔN in EcoRI-XbaI sites of pET28a | This study |
| pMV261 | Kan^r^, pAL5000 replicon, lacZ-fusion plasmid backbone | [3] |
| pMZ0 | Kan^r^, lacZ fusion with none promoter | This study |
| pMZ+ | Kan^r^, lacZ fusion with hsp60 promoter | This study |
| pMZ1 | Kan^r^, lacZ fusion with Mt2034p | This study |
| pMZ2 | Kan^r^, lacZ fusion with Mt2034p and Rv2034 | This study |
| pMZ3 | Kan^r^, lacZ fusion with Mt2034p and Rv2034AE | This study |
| pMZ4 | Kan^r^, lacZ fusion with Mt2034p and Rv2034C61A | This study |
| pMZ5 | Kanr, lacZ fusion with Mt2034p and Rv2034ΔN | This study |
| pMZ6 | Kanr, lacZ fusion with Mt3133cp | This study |
| pMZ7 | Kanr, lacZ fusion with Mt3133cp and Rv3133c | This study |
| pMZ8 | Kanr, lacZ fusion with Mt3133cp and Rv2034 | This study |
| pMZ9 | Kanr, lacZ fusion with Mt3133cp, Rv3133c, andRv2034 | This study |
