# Supplementary material for: Characterization of a Novel ArsR-Like Regulator Encoded by Rv2034 in Mycobacterium tuberculosis
Source: PLoS One. 2012 Apr 27;7(4):e36255. doi: 10.1371/journal.pone.0036255 (PMC3338718; doi:10.1371/journal.pone.0036255)
Supplement: Table S3 — DNA substrates used in this study. (DOC) [file pone.0036255.s006.doc]

## Supplemental Table 3

DNA substrates used in this study

| **Name** | **Length and Source** | **Sequence or primers used to amplify long segments (5’–3’)** |
| --- | --- | --- |
| Rv2034p | ~250bp, PCR | Mt2034pf: AATTTCTAGAGCCTGCGGCAAGCACGTCGG  Mt2034pr: CCGGGAATTCGAACCGTAAGTTTAGACTTA |
| GFP | ~700bp, PCR | GFPf-Hind3: ATGCAAGCTTATGGTGAGCAAGGGCGAGGA  GFPr-NheI: ATCAGCTAGCCTTGTACAGCTCGTCCATGC |
| Rv3133cp | ~250bp, PCR | Mt3133cpf: ACTGTCTAGAGTTGGCGCATGTACACCTGAGCCGT  Mt3133cpr: AGATGAATTCCAGGGCACCACTCCCAAGATCCGCT |
| Rv2034p  FITC labeled (+) | ~220bp, PCR | Mt2034p-140f-FITC: GATAGAATTCTACCAGTCGACTTTCCGGCG  Mt2034+82r: GACATCTAGATGCGCCAGCCGCTCCACGAT |
| Rv2034p  FITC labeled (-) | ~220bp, PCR | Mt2034p-140f: GATAGAATTCTACCAGTCGACTTTCCGGCG  Mt2034+82r-FITC: GACATCTAGATGCGCCAGCCGCTCCACGAT |
| Rv2034BSwt | 40bp, synthesized | 5’-GGAAGAATATCCGTAAGTCTAAACTTACGGTTCGTGTCCA-3’ |
| Rv2034BSmu1 | 40bp, synthesized | 5’-GGAAGAATATGGCATTCACTAAACTTACGGTTCGTGTCCA-3’ |
| Rv2034BSmu2 | 40bp, synthesized | 5’-GGAAGAATATGGCATTCACTAAACTGCATGTTCGTGTCCA-3’ |
| Rv2034BSmu3 | 44bp, synthesized | 5’-GGAAGAATATCCGTAAGTCTGATAAAACTTACGGTTCGTGTCCA-3’ |
